# Mapping of Early Exercise‐Based Interventions for Patients Recovering From Acute Heart Failure: A Scoping Review

**DOI:** 10.1161/JAHA.125.045954

**Published:** 2026-02-11

**Authors:** Akhila Satyamurthy, Ramachandran Padmakumar, Sivadasanpillai Harikrishnan, Panniyammakal Jeemon, Mukund A. Prabhu, Abraham Samuel Babu

**Affiliations:** ^1^ Department of Physiotherapy Manipal College of Health Professions, Manipal Academy of Higher Education Manipal Karnataka India; ^2^ Department of Cardiology Kasturba Medical College, Manipal Academy of Higher Education Manipal Karnataka India; ^3^ Department of Cardiology Sree Chitra Tirunal Institute of Medical Sciences & Technology Thiruvananthapuram Kerala India; ^4^ Achutha Menon Centre for Health Science Studies, Sree Chitra Tirunal Institute of Medical Sciences & Technology Thiruvananthapuram Kerala India

**Keywords:** acute heart failure, cardiac rehabilitation, early mobilization, Heart Failure, Exercise

## Abstract

Studies have established the safety and benefits of exercise training in patients admitted with acute heart failure, yet heterogeneity in exercise delivery patterns exists. Hence, a scoping review was undertaken to map the evidence on early exercise‐based interventions (early mobilization with/without exercise training) in patients recovering from acute heart failure (admission to up to 2 weeks post‐hospitalization), for geographic distribution, exercise prescription, and exercise initiation time. A systematic search was conducted across 5 databases until September 2024. Studies, including protocols, providing early exercise‐based intervention anytime between admission and up to 2 weeks from discharge, in any setting, were included. Data were extracted from 30 included studies, and the obtained evidence was mapped. This study uses the Preferred Reporting Items for Systematic Reviews and Meta‐analyses (PRISMA)—Extension for Scoping Reviews. The review included 1,54 980 participants with acute heart failure, and 26.6% (8 of 30) of the studies focused on the older adult population. Early exercise‐based interventions, ie, early mobilization (n=12), exercise training (n=5), or combined (n=13), were limited to higher‐ (n=23) and upper‐middle‐income (n=6) countries and were primarily observational (n=19) in design. The median (Q1‐Q3) initiation time to exercise was 3.8 days (2.8–5.5), with a dose of eight sessions (4.7–21). The intensity ranged from very low to moderate intensity, with the duration per session ranging from 10 to 60 minutes. The use of pre‐specified, well‐developed initiation, monitoring, and termination criteria was not common. Early exercise‐based interventions were comprehensive, multi‐modal, and of low‐moderate intensity, initiated within 4 days of admission.

**Nonstandard Abbreviations and Acronyms**
AHF: acute heart failure
CR: cardiac rehabilitation
LMIC: lower‐middle income country
RCT: randomized controlled trial
6MWT: 6‐minute walk test
RPE: rating of perceived exertion
JCS: Japanese Cardiology Society

Acute heart failure (AHF) is defined as “rapid onset or worsening of symptoms and/or signs of heart failure (HF)”.^1^ It is associated with high hospital readmission rates of >50%, especially among older people, resulting in 5% emergency admissions and an in‐hospital mortality rate of 5% to 11%.^2^ Patients admitted with AHF experience malnutrition, impaired mobility, and increased difficulty in performing daily activities, all of which worsen with every readmission.^3^, ^4^ Moreover, AHF is characterized by an unpredictable recovery trajectory, predisposing patients to prolonged hospitalization and repeat hospitalization.^5^

Acute onset of HF symptoms is a contraindication for exercise training, and patients with AHF are identified as a high‐risk group for exercise training.^6^ It is reflected in the literature, as exercise training is initiated at discharge or within a few days of discharge instead of during hospitalization.^7^ Nevertheless, the recovery period and early post‐discharge period (within 2–14 days of hospital discharge) are considered a golden opportunity to improve patient‐related outcomes.^5^ Delivering timely, evidence‐based exercise interventions—comprising early mobilization and structured exercise training—during this phase may enhance recovery, reduce hospital stay, and promote sustained participation in rehabilitation after discharge. However, the inherent clinical nature of AHF could limit the utilization of exercise interventions, even though early mobilization reduces hospital stay.^8^

The growing prevalence of HF in older people and associated functional decline in patients with AHF was the driving force to assess the importance of early exercise training to improve short‐term and long‐term outcomes.^4^ The landmark Rehabilitation Therapy in Older AHF patients (REHAB‐HF) trial, which initiated a progressive, individualized multi‐domain rehabilitation program, found it beneficial in terms of function for older patients with AHF.^9^ Recent reviews have identified the literature on exercise training in patients with AHF.^10^, ^11^, ^12^ Systematic reviews with/without meta‐analysis have also established the safety and benefits of early exercise training on functional outcomes and re‐hospitalization rates.^7^, ^10^, ^11^, ^13^ However, more than half (5 of 9) of the studies initiating exercise during hospitalization were non‐randomized, leading to their exclusion from quantitative syntheses.^7^ Furthermore, one review identified the ongoing trials to have heterogeneity and, in some instances, ambiguity with respect to the initiation time of cardiac rehabilitation (CR), choice of interventions, and monitoring checklist and criteria.^7^

Given this variability in implementation across health care systems, we undertook a scoping review to organize and map the existing early exercise‐based interventions (early mobilization with/without exercise training) in patients recovering from AHF (from admission to up to 2 weeks from discharge). Specifically, we sought to describe the geographic distribution, timing of initiation, and key characteristics of these interventions, including frequency, intensity, time, type, and dosage. This synthesis aimed to elucidate variations in practice and identify evidence gaps to inform future research and clinical implementation strategies.

## Methodology

### Study Design

It was a scoping review with intervention mapping, with no a priori published protocol. This review followed the framework of Arksey and O’Malley^14^ and was reported using the Preferred Reporting Items for Systematic Reviews and Meta‐analyses (PRISMA)—Extension for Scoping Reviews (Table S1).^15^

### Search Strategy

A search was conducted across 5 databases: PubMed, MEDLINE, Embase, Scopus, and Web of Science, using the strategy summarized in Table S2. The search was initiated on 6th July 2021 and was updated every year until September 1, 2023. Keywords such as AHF, CR, exercise training, and their respective Medical Subject Headings terms were used, along with the Boolean operators’ ‘OR’ and ‘AND’. After the review was completed, the search was updated till 21st September 2024.

### Inclusion Criteria

The review included studies on individuals admitted with AHF (defined as patients with signs of decompensation, either de novo or decompensated, who required hospital admission) who participated in early exercise‐based interventions (early mobilization with/without early exercise training), anytime between admission and up to 2 weeks from discharge, in any setting. Considering the aim of the study, the authors did not limit inclusion to any specific study design and included study protocols. Study protocols were used for both qualitative and quantitative evidence synthesis.

### Exclusion Criteria

Studies were excluded based on type of study design (review papers), timing of intervention (at discharge and initiated intervention beyond 2 weeks from discharge), type of population (participants other than AHF, studies that specified patients to be stable with no change in medications), and type of intervention (interventions focused only on patient education, electrical muscle stimulation, and non‐invasive ventilation, and studies in which exercise was not the focus of the study). Animal studies and non‐English studies were excluded. Studies that were also excluded.

### Data Extraction

Articles were screened for duplicates and eligibility criteria using the Rayyan Software^16^ by AS and ASB. Conflicts that arose during the process were resolved by RP. AS manually extracted data on a standardized Excel sheet, followed by verification of the extracted data by ASB and resolution of any disputes by RP. We extracted citation details (author names, year of publication, and country where the study was conducted), study characteristics (study design and sample size), study population characteristics (age, sex, type of acute heart failure mainly de novo and/or decompensated, HF phenotype, and New York Heart Association class), inclusion and exclusion criteria, intervention characteristics (frequency, intensity, duration, type, time to initiation, safety criteria, termination criteria, exercise dose), and physical function related outcome measures. Authors of the included study were contacted for additional information, and in cases where no response was received, despite 2 reminders, the data were considered missing. In this review, early mobilization was defined as gradual mobilization from supine position to walking with/without in‐bed exercises and out‐of‐bed exercises. Early exercise training was defined as a structured exercise program across the domains of aerobics, resistance, and/or balance.

### Evidence Mapping

**Figure 1:**
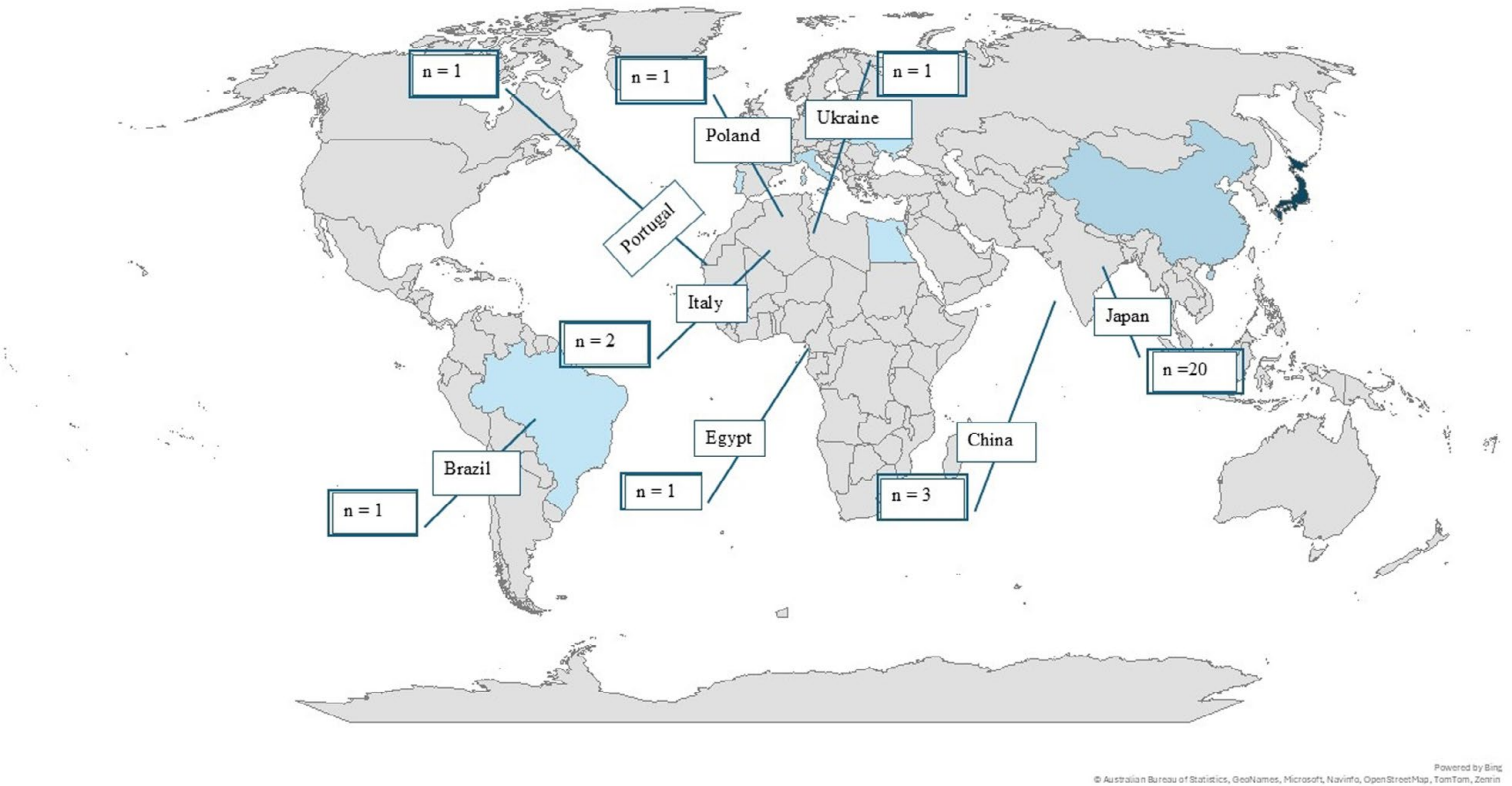
Geographic distribution of included studies (n=30). This figure shows the geographical spread of where the studies were conducted. Lighter shades indicate the least number of studies being conducted in that country (eg, Brazil). Darker shades indicate highest number of studies being conducted in that country (eg, Japan).

**Figure 2:**
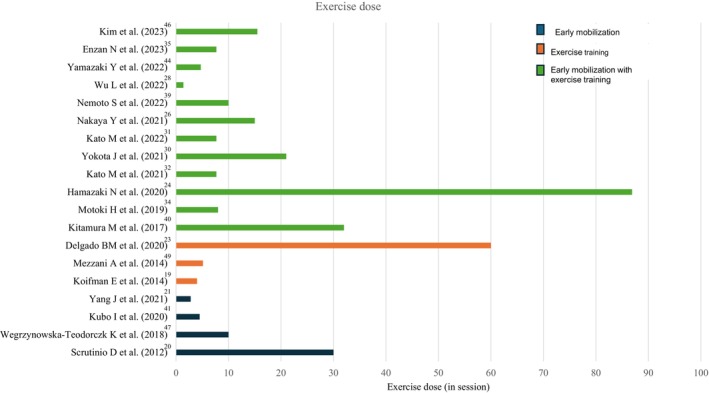
Mapping of exercise dose (n=19). This figure plots the exercise dosage used in studies that provided exercise training individually or in combination. Exercise dose was calculated as the average number of sessions per week multiplied by the number of weeks.

**Figure 3:**
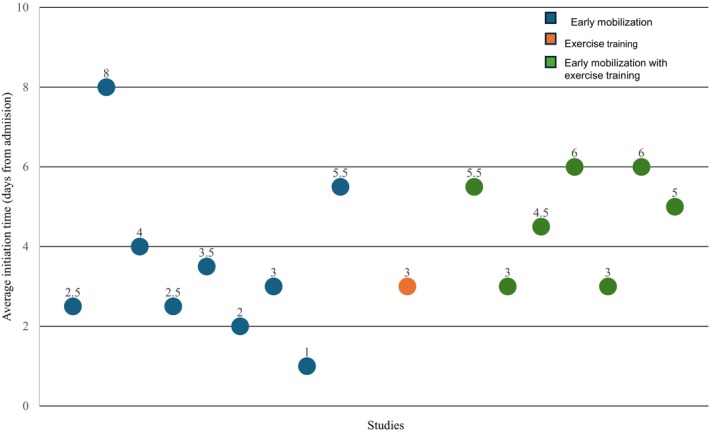
Mapping the initiation time for early exercise‐based intervention (n=18). This figure depicts the average time taken (from admission) to initiate early exercise‐based intervention. Each bubble represents a single study plotted in the ascending order of the year of publication.

We used evidence mapping to describe the nature of existing early exercise‐based interventions.^17^ The data were reorganized and plotted into 3 mapping figures using Microsoft Excel: (1) a location map to understand the geographical distribution of the studies and their settings (Figure 1), (2) a bar graph to project the dosage of early exercise training (Figure 2), and (3) a bubble plot to describe the initiation time (Figure 3). Figure 1 shows the country in which the study was conducted. Countries were classified according to the World Bank 2024 to 2025 country classification into 4 income groups: high‐income, upper‐middle‐income, lower‐middle‐income, and low‐income. The exercise dose was calculated as the average number of sessions per week multiplied by the number of weeks (Figure 2).^18^ Figure 3 was obtained by plotting the average time (in days) taken to initiate early exercise‐based interventions from the day of admission. To plot Figure 3, we calculated the average initiation time for studies that provided a range for initiation time (Table 2). For example, the initiation time reported as 3 to 8 days from admission was calculated as 3+8/2=5.5 days. Studies that reported the initiation time as a mean value, a median, and a single numerical value were plotted as shown in Figure 3. Studies that reported the initiation time from discharge, and did not specify the initiation time (Table 2), were excluded from Figure 3.

## Results

In total, 7779 articles were obtained from the databases with the search strategy summarized in the (Table S2). After the completion of the updated search, a total of 30 full‐text articles were included in the scoping review, Figure S1. Studies were excluded based on the type of population (n=20), type of paper (ie, review papers) (n=9), type of intervention (n=13), timing of intervention (n=16), and others (n=43). Data from 19 articles were reorganized to plot the 3 evidence maps.

### Characteristics of Included Studies

A total of 154 980 (n=30 studies) AHF participants, both with de novo and decompensated chronic HF, were included in this review. The study population comprised adult participants (>18 years), and 26.6% (8 of 30) of the studies focused on the older adult population. Only one study focused on HF with preserved EF.^19^

Overall, the 2 most frequently used outcome measures were the 6‐minute walk test, 33.3% (10 of 30)^19^, ^20^, ^21^, ^22^, ^23^, ^24^, ^25^, ^26^, ^27^, ^28^ and the Barthel index (BI), 33.3% (10 of 30).^23^, ^25^, ^29^, ^30^, ^31^, ^32^, ^33^, ^34^, ^35^, ^36^, ^37^ Interestingly, studies on early mobilization prioritized functional outcome measurements over formal assessments of exercise capacity (Table 3). The effects of early mobilization on the ability to walk were measured in terms of exertional dyspnea (dyspnea upon walking 10 meters measured using the visual analogue scale, and New York Heart Association grade), distance covered (eg, the walking level before discharge), time taken (time to achieve more than 40 meters or 50 meters independently or early attainment of walking), in addition to lower limb endurance (2 minute step test and 30 second chair stand test), and the ability to perform activities of daily living (BI, motor functional independence measure, and the Rivermead mobility index). However, studies on exercise training alone or in combination have measured functional and exercise capacity using the 6‐minute walk test, cardiopulmonary exercise testing, and/or short physical performance battery (Table 3).

### Geographical Distribution

Studies on AHF have been conducted in upper‐middle income and higher‐income countries, which were spread across China (n=3), Japan (n=20), the USA (n=1), Africa (n=1), and various European countries (n=5), with Japan leading the number of studies (Figure 1). There was only 1 study conducted in a lower‐middle‐income country (LMIC).^27^ Overall, the study designs were diverse and included 19 observational studies [6 prospective^20^, ^24^, ^25^, ^30^, ^38^, ^39^ and 13 retrospective studies^29^, ^32^, ^33^, ^34^, ^35^, ^37^, ^40^, ^41^, ^42^, ^43^, ^44^, ^45^, ^46^] 9 randomized controlled trials (RCTs),^19^, ^23^, ^26^, ^27^, ^28^, ^36^, ^47^, ^48^, ^49^ 1 quasi‐experimental study,^21^ and 1 secondary data analysis (Table 1).^32^

**Table 1:** Characteristics of the Included Studies

Sl. No.	Author (year)	Study design	Location	Sample size	Population	Type of intervention*	Initiation time	Total duration of exercise
1.	Scrutinio D et al. (2012)^20^	Prospective observational	Italy	275	ADHF	CR†	4 d from DC	6–30 d
2.	Mezzani A et al. (2014)^49^	RCT	Italy	200	ADHF	Early aerobic exercise training	3 d from admission	12 d
3.	Kitamura M et al. (2017)^40^	Retrospective observational	Japan	589	>65 y and admitted HF	Early mobilization and aerobic training	Unclear	10–20 d
4.	Wegrzynowska‐Teodorczk K et al. (2018)^47^	RCT (protocol)	Poland	42	AHF	CR† (experimental group) vs standard pharmacotherapy (control group)	2–3 d from admission	4 weeks
5.	Kakutani N et al. (2019)^33^	Retrospective observational	Japan	136	De novo AHF and ADHF	Early mobilization (experimental group) vs usual care (control group)	2–14 d from admission	2–48 d
6.	Kubo I et al. (2020)^41^	Retrospective observational	Japan	180	>65 y and admitted HF	Early mobilization	1–7 d from admission	8–39 d
7.	Motoki H et al. (2019)^34^	Retrospective observational	Japan	171	ADHF	CR†	3–8 d from admission	2 weeks
8.	Kitamura M et al. (2019)^42^	Retrospective observational	Japan	377	>65 y and admitted HF	Early mobilization	0–5 d from admission	8–30 d
9.	Delgado BM et al. (2020)^23^	RCT	Portugal	100	ADHF	ERIC‐HF protocol (experimental group) vs ACSM protocol (control group)	Unclear	5–41 d
10.	Hamazaki N et al. (2020)^24^	Prospective observational	Ukraine	456	Admitted HF	CR†	3 d from admission	5 mo
11.	Takada S et al. (2020)^29^	Retrospective observational	Japan	259	>65 y and ADHF	Early mobilization	3–4 d from admission	Unclear
12.	Yokota J et al. (2021)^30^	Prospective observational	Japan	294	AHF	CR†	2–7 d from admission	60 d
13.	Yang J et al. (2021)^21^	Quasi‐experimental (interrupted time series)	China	110	AHF	Early mobilization	Unclear	7–11 d
14.	Kato M et al. (2021)^32^	Secondary analysis	Japan	411	>60 y and AHF	CR†	3–9 d from admission	5–12 d
15.	Shibata A et al. (2021)^38^	Prospective observational	Japan	94	ADHF	CR†	Unclear	DC
16.	Sakai T et al. (2021)^43^	Retrospective observational	Switzerland	220	AHF and ADHF	CR†	Hemodynamically stable with or without IV diuretics	Unclear
17.	Nemoto S et al. (2022)^39^	Prospective cohort	Japan	247	ADHF	CR†	3 d from admission	DC
18.	Wu L et al. (2022)^28^	RCT (protocol)	China	120	Admitted ADHF	CR†	Unclear	5 d
19.	Kato M et al. (2022)^31^	Retrospective observational	Japan	956	>60 y and ADHF	CR†	3–9 d from admission	13–28 d
20.	Yamazaki Y et al. (2022)^25^	Prospective observational	Japan	54	Admitted HF	Early mobilization	0–4 d from admission	6–11 d
21.	Yamazaki Y et al. (2022)^44^	Retrospective observational study (letter)	Japan	375	>80 y and ADHF	Early mobilization (those who received vs who did not receive CR†)	Unclear	DC
22.	Nakaya Y et al. (2021)^26^	RCT	Japan	75	>60 y and ADHF	CR† with multi‐faceted exercise training (experimental group) vs CR† only (control group)	5 d from admission	DC
23.	Ahmad MA et al. (2023)^27^	RCT	Egypt	30	ADHF	Early mobilization (intervention group) vs standard medical care (control group)	3 d from admission	DC
24.	Funato Y et al. (2022)^45^	Retrospective observational	Japan	560	ADHF	Acute phase ambulation program vs those who did not receive it	Hemodynamic stabilization	DC
25.	Wang Y et al. (2022)^48^	RCT	China	98	AHF with coronary heart disease	CR† (experimental group) vs standard medical care (control group)	As and when they satisfy the initiation criteria	7 d
26.	Enzan N et al. (2023)^35^	Retrospective observational	Japan	10 473	ADHF	CR†	Hemodynamic stabilization	DC
Early exercise intervention in patients with HFpEF								
27.	Koifman E et al. (2014)^19^	RCT (protocol)	Japan	1224	Stabilized acute HFpEF	Multidisciplinary CR† including exercise training (experimental group) vs usual care (control group)	Within 2‐4 week after DC	6 mo
Updated search (till 21st September, 2024)								
28.	Kim J et al. (2023)^46^	Retrospective observational	Japan	88 052	AHF	CR† ^,^ ‡	4.6 ± 5.5 d from admission	18.6 d
29.	Fraga IB et al. (2023)^36^	RCT (protocol)	Brazil	54	ADHF	Early mobilization	24 h from admission	1–3 d
30.	Ishibashi T et al. (2023)^37^	Retrospective observational	Japan	48 748	Admitted HF	Early mobilization	5 (3‐8)d from admission	DC

ADL indicates activities of daily living; ADHF, acute decompensated heart failure; AHF, acute heart failure; AT, atrial tachycardia; BI, Barthel index; BMI, body mass index; CR, cardiac rehabilitation; CHQc, chronic heart failure questionnaire Chinese; CONUT, controlling nutritional status; DC, discharge; d‐ROM, diacron reactive oxygen metabolites; d, days; DASI, Duke activity status index; EMS, electrical muscle stimulation; eGFR, estimated glomerular filtration rate; ET, exercise training; FOIS, functional oral intake scale; FIM, functional independence measure; HR, hazards ratio; HF, heart failure; HFpEF, heart failure with preserved ejection fraction; IRR, incident rate ratio; IADL, instrumental activity of daily living; IV, intravenous; LCADL, London chest activity of daily living; MD, mean difference; MLHFQ, Minnesota living with heart failure questionnaire; mo, months; NMES, neuromuscular electrical stimulation; NIV, non‐invasive ventilation; NA, not applicable; Unclear, not clear; OR, odds ratio; SPO2, oxygen saturation; peakVO2, peak oxygen uptake; PImax, maximal inspiratory pressure; QIS, quadriceps isometric strength; RCT, randomized controlled trial; RPE, rating of perceived exertion; RR, respiratory rate; RMI, Rivermead index; 6MWD, 6‐minute walk distance; SPPB, short physical performance battery; UHT, urgent heart transplantation; and VAS, visual analogue scale.

* Detailed description of the intervention is given in Table 2.

^†^ CR indicates early mobilization in combination with exercise training.

^‡^ The study focused on both in‐patient and out‐patient CR, but the table discusses details of only in‐patient CR.

### Timing and Initiation of Early Exercise‐Based Interventions

Early exercise‐based intervention was initiated at a median (quartile 1—quartile 3) of 3.8 days (2.8–5.5) from admission (Table 1). Figure 3 displays both heterogeneity and delays in the initiation time of early mobilization. However, early mobilization combined with exercise training was initiated uniformly between 3 and 6 days after admission. Only 1 study began the exercise intervention within 24 hours^36^ (Figure 3), and only 1 study used a criterion to initiate exercise training, ie, (1) no chest pain in the past 8 hours, (2) no evident symptoms or signs of decompensated heart failure, (3) no new onset arrhythmia nor changes on electrocardiograph (ECG), and (4) no elevation of NT‐proBNP.^48^

All included studies^19^, ^20^, ^21^, ^23^, ^24^, ^25^, ^26^, ^27^, ^28^, ^29^, ^30^, ^31^, ^32^, ^33^, ^34^, ^35^, ^36^, ^37^, ^38^, ^39^, ^40^, ^41^, ^42^, ^43^, ^44^, ^45^, ^46^, ^47^, ^49^, ^50^ highlighted the need to establish hemodynamic stability, as determined by health care professionals, before the initiation of early exercise‐based interventions. However, they lacked structured criteria that define ‘hemodynamic stability’.

### Exercise Prescription (Frequency, Intensity, Time, Type, and Dose)

After mapping the literature for the choice of intervention delivered to patients with AHF during the recovery phase (Table 2), early mobilization with structured exercise training was the most commonly used intervention (12 of 30, 40%). Irrespective of the type of exercise intervention, the exercise dose varied across studies, with a median exercise training dose of 8 sessions (4.7–21) (Figure 2). The exercise prescription for each intervention is described below in terms of dosage, frequency, intensity, and type.

**Table 2:** Characteristics of the Intervention

Sl. No.	Author (year)	Frequency	Intensity	Time (per session)	Type (mode of exercise)	Exercise dose (average number of sessions/week×number of weeks*)
Early mobilization						
1.	Scrutinio D et al. (2012)^20^	2–3 sessions daily, 5 times/wk for 20.8 ± 14.1 d	Low intensity, individualized	15–30 min	Stationary cycling	5×6=30 sessions
2.	Wegrzynowska‐Teodorczk K et al. (2018)^47^	2–3 times/wk for 4 wk	Very low intensity, short duration, with recovery phase and individualized	10 min and progressed to 20–25 min	Active dynamic exercise, assisted exercises, flexibility exercise and body‐weighted resisted exercise (in all positions of lying, sitting and standing)	2.5×4=10 sessions
3.	Kakutani M et al. (2019)^33^	Number of sessions: unclear Total duration: 2–48 d	Mobilization: not mentioned Aerobic and balance exercise: 12–13/20 Borg’s RPE to maintain 2.3–2.5 METs Resisted exercise: <13/20 on Borg’s RPE	Not mentioned	Active range of motion exercise, 7‐staged gradual mobilization, and Aerobic exercise: walking or cycle ergometer Resisted exercise: dumb‐bells and weighted machines	Unable to calculate
4.	Kubo I et al. (2020)^41^	Frequency as per patient symptoms and physical assessment Total duration: 1 wk	As per patient symptoms and physical assessment	Aerobic exercise: 50–80 min for 5–10 min or on a bicycle ergometer at 10–20 W for 5–10 min Resisted exercise: RPE<13/20	Gradual mobilization with aerobic exercise^†^: walking or bicycle ergometer Resisted exercise: rubber band, weights, dumb‐bell, free weights	Aerobic exercise: 2×1=2 sessions Resisted exercise: 2.5×1=2.5 sessions
5	Kitamura M et al. (2019)^42^	Frequency as per patient symptoms and physical assessment Total duration: 8–30 d	As per patient symptoms and physical assessment	Aerobic exercise: 50–80 min for 5–10 min or on a bicycle ergometer at 10–20 W for 5–10 min	Gradual mobilization with aerobic exercise†: walking or bicycle ergometer	8×4=32 for aerobic exercises
6.	Takada S et al. (2020)^29^	Frequency as per patient symptoms and physical assessment Total duration: unclear	As per patient symptoms and physical assessment	Aerobic exercise: 50–80 min for 5–10 min or on a bicycle ergometer at 10–20 W for 5–10 min Resisted exercise: RPE<13/20	Gradual mobilization with aerobic exercise^†^: walking or bicycle ergometer Resisted exercise: rubber band, weights, dumb‐bell, free weights	Aerobic exercise: 2×1=2 sessions Resisted exercise: 2.5×1=2.5 sessions
7.	Yang J et al. (2021)^21^	2 times/d with each movement lasting for 5–6 seconds for 7–11 d	Mobilization stage and exercises were chosen based on NYHA class	10–20 min	NA	2×1.4=2.8 sessions
8.	Yamazaki Y et al. (2022)^20^	3 times/d for 6–11 d	Borg’s RPE=13/20	Not given	NA	3×1.57=4.71 sessions
9.	Ahmad MA et al. (2023)^27^	2 times/d Total duration: unclear	Stages 1–3: THR=resting HR +20 bpm and Borg’s RPE=11–12/20 Stage 4: THR=resting HR+20‐30 bpm and Borg’s RPE ≤13 Stage 5: THR=resting HR +30 bpm and Borg’s RPE ≤13	Stage 1–3: <10 min Stage 4: 5–10 min Stage 5: 5–15 min	Stage 1–2: active exercises for all 4 limbs in all positions from supine to standing with strengthening exercise in Stage 1 and balance exercise in Stage 2 Stage 3–4: Endurance exercise in the form of walking Stage 5: walking and stair climbing	Unable to calculate
10.	Funato Y et al. (2022)^45^	Unclear	Based on patient’s hemodynamic response to exercise loading test	Not given Target sitting time per day: 1–3 h	Stage 1–6 included gradual mobilization† from bed rest to walking within ward	Unable to calculate
11.	Fraga IB et al. (2023)^36^	Unclear	Patient’s tolerance measured using Borg’s scale, vitals response and response to the question “In your opinion, were you able to tolerate this exercise well?”	5–10 min/session	Mobilization with in‐bed cycle ergometry for upper limb and lower limb	1–3 sessions
12.	Ishibashi T et al. (2023)^37^	Unclear				
Exercise training						
13.	Koifman E et al. (2014)^19^	2 times/wk for 6 mo	Aerobic exercise: 40%‐50% HRR for those who tolerate stress test with RPE<13/20 and THR goal 20–30 beats above baseline Resisted exercise: 10–12 reps at Borg’s RPE<14/20	10+15+10=35 min	Aerobic exercise: treadmill, bicycle, inclined stepper Resisted exercise using weights, elastic band, and balls	2×2=4 sessions
14.	Mezzani A et al. (2014)^49^	3 sessions/d for 12 d	Low intensity Cycle ergometer progressed from unloaded cycling to10‐20 W	30+20=50 min	Aerobic exercise: cycle ergometer along with assisted ambulation	3×1.71=5.13 sessions
15.	Delgado BM et al. (2020)^23^	2 times/d×5 d/wk=10 sessions/wk for 5–41 d	Modified Borg’s RPE<8/10	5–20 min	Walking, bicycle ergometer	10×6=60 sessions
16.	Shibata A et al. (2021)^38^	5 times/wk Total duration: unclear	Anerobic threshold determined by CPx, using the v‐slope method. For patients who were unable to perform CPx, intensity was set using Karvonen’s formula with Karvonen’s co‐efficient set at 0.4–0.6	30 min	Not mentioned	Unable to calculate
17.	Wang Y et al. (2022)^48^	Not given Total duration: 1 wk	20 ± 5 beats rise from the resting heart rate and Borg’s RPE	30 min	Aerobic exercise with warm up: modality not mentioned Meridians patting or flexibility training	Unable to calculate
Early mobilization with exercise training						
18.	Kitamura M et al. (2017)^40^	Early mobilization: NA Exercise training: Aerobic exercise: 3–5 times/d Resisted exercise: 2–3 times/wk Total duration: unclear	Early mobilization: 11–13/20 with a target HR 20‐30 bpm rise from baseline and resisted: Borg’s RPE<13/20 Aerobic exercise: 40%–60% HRR or Borg’s RPE=11–13/20 Resisted exercise: 30%–40% of 1 RM for UL and 50%–60% of 1RM for LL or Borg’s RPE<13/20, 1–3 sets, 10–15 times	Early mobilization: begin with 5–10 min (progression is individualized) Aerobic exercise: 5–10 min (progress to 10–30 min)	Early mobilization: walking or cycle ergometer Aerobic exercise: walking or cycle ergometer	NA
19.	Motoki H et al. (2019)^34^	5 times/wk For severe patients: 3 times/wk for 2 wk	Early mobilization: not mentioned Exercise training: Karvonen intensity value of 0.3–0.5 or Borg RPE of 11–13/20	Early mobilization: not mentioned Aerobic exercise: 30 min	Early mobilization: NA Aerobic exercise: walking or bicycle ergometer Resisted exercise: squats, calf raises and weight training	4×2=8 sessions
20.	Hamazaki N et al. (2020)^24^	Unclear	Low to moderate intensity exercise training for patients who cover 200 m with independence	Unclear	Early mobilization† ^,^ §	NA
21.	Yokota J et al. (2021)^30^	60 d	Low intensity resisted and aerobic exercise training	Aerobic exercise: 50–80 min for 5–10 min or on a bicycle ergometer at 10–20 W for 5–10 min Resisted exercise: RPE<13/20	Gradual mobilization with aerobic exercise^†^: walking or bicycle ergometer Resisted exercise: rubber band, weights, dumb‐bell, free weights	21 sessions
22.	Kato M et al. (2021)^32^	4–5 times/wk for 5–12 d	Early mobilization Endurance training: Borg’s RPE=11–13/20 or 40%–60% HRR Resisted exercise: Borg’s RPE<13/20 (UL=30%–40% of 1RM and LL=50%–60% of 1RM)	20–60 min	Early mobilization^†^ ^,^ ^‡^: NA Endurance training: stationary bike or treadmill Resisted exercise: rubber band, muscle training machine or body‐weighted	4.5×1.71=7.69 sessions
23.	Sakai T et al. (2021)^43^	3–5 times/wk for walking and 2‐3 times/wk for exercise training Total duration: unclear	Early mobilization with exercise training Endurance training: Borg’s RPE=11–13/20 or 40%–60% HRR Resisted exercise: Borg’s RPE<13/20 (UL=30%–40% of 1RM and LL=50%–60% of 1RM)	5–10 min and progressed to 10–30 min	Early mobilization^†^ to standing and walking Aerobic exercises via cycle ergometer	NA
24.	Nemoto S et al. (2022)^39^	5 times/wk for 20 d	Unstable stage: RPE<12 or ≤30% of 1RM Stable stage: RPE=12–13 or 30%–40% of 1RM	20–60 min	Early mobilization: NA Aerobic exercise: cycle ergometer or treadmill Resisted exercise: not mentioned	5×2=10 sessions
25.	Wu L et al. (2022)^28^	2 times/day for 5 d	Level 1–2=5–15 HR increase, Borg’s RPE <12, Perme score=3 Level 3–7=20–30 HR increase, Borg’s RPE=12–13/20, Perme score=3	Sitting=2–5 min Standing=2–5 min Walking=2 min Aerobic exercise=10–20 min Progressive resisted exercise=15 times for each muscle group, 3 muscle groups with 2‐min rest between each muscle group	Early mobilization: NA Aerobic exercise: bedside treadmill Resisted exercise: body‐weighted exercises	2×0.7=1.4 sessions
26.	Kato M et al. (2022)^31^	4–5 times/week for 5–12 d	Early mobilization Endurance training: Borg’s RPE=11–13/20 or 40%–60% HRR Resisted exercise: Borg’s RPE<13/20 (UL=30%–40% of 1RM and LL=50%–60% of 1RM)	20–60 min	Early mobilization^†^ ^,^ ^‡^: NA Endurance training: stationary bike or treadmill Resisted exercise: rubber band, muscle training machine or body‐weighted	4.5×1.71=7.69 sessions
27.	Yamazaki Y et al. (2022)^44^	Treatment details not available				
28.	Nakaya Y et al. (2021)^26^	5 times/wk Total duration: unclear	Early mobilization: not mentioned Aerobic training: interval training with work time of 30–60 s followed by recovery in the ratio 1:2 Resisted training: 5 reps×2 sets at 11–12/20 Borg’s RPE Balance training: 11–12/20 Borg’s RPE with 10 s for each exercise	20–40 min	Early mobilization^†^: NA Aerobic training: cycle ergometer Resisted training: closed chain body‐weighted exercises and open chain with weights Balance training: NA	5×3=15 sessions
29.	Enzan N et al. (2023)^35^	4–5 times/wk Total duration: unclear	Early mobilization Endurance training: Borg’s RPE=11–13/20 or 40%–60% HRR Resisted exercise: Borg’s RPE<13/20 (UL=30%–40% of 1RM and LL=50%–60% of 1RM)	20–60 min	Early mobilization^†^ ^,^ ^‡^: NA Endurance training: stationary bike or treadmill Resisted exercise: rubber band, muscle training machine or body‐weighted	NA
30.	Kim J et al. (2023)^46^	3–5 d/wk Total duration: 12.4 d	Unclear	Unclear	Early mobilization^†^ ^,^ ^§^ ^,^ ^||^: Exercise training^†^ ^,^ ^§^: stretching, resistance, and aerobic training	15.5 sessions

bpm indicates beats per minute; BiPAP, bilevel positive airway pressure; CPx, cardiopulmonary exercise test; min, minute; EMS, electrical muscle stimulation; HR, heart rate; HRR, heart rate reserve; Hz, hertz; JCS, Japanese Cardiology Society; LL, lower limb; METs, metabolic equivalents; m, meter; NA, not applicable; NMES, neuromuscular electrical stimulation; NYHA, New York Heart Association; VO2peak, peak oxygen uptake; RPE, rating of perceived exertion; reps, repetitions; RM, repetition maximum; sec, seconds; THR, target heart rate; UL, upper limb; and W, watt.

* Studies which reported the total duration of intervention in days were converted into weeks by dividing it by 7.

^†^ Intervention was delivered as per the guidelines by the Japanese Circulation Society.

^‡^ Intervention was delivered as per the guidelines by Japanese Association of Cardiac Rehabilitation.

^§^ This study provided in‐hospital phase CR and out‐patient CR. This table only describes the in‐hospital phase CR.

^||^ Intervention was delivered as per the American College of Sports Medicine’s guidelines for exercise testing and prescription.

**Table 3:** Characteristics of Physical Function Related Outcome Measures

Sl. No.	Author (year)	Functional outcomes	Time‐points of measurement
Early mobilization			
1.	Scrutinio D et al. (2012)^20^	6MWT NYHA class	At admission and discharge (median of 219 d) At admission
2.	Wegrzynowska‐Teodorczk K et al. (2018)^47^	2‐min step test, 30 s “chair stand” test, “up and go” test, and hand‐grip strength	At discharge and 30 d
3.	Kakutani N et al. (2019)^33^	BI	At discharge
4.	Kubol I et al. (2020)^41^	Walking level before hospitalization Motor FIM Early acquisition of walking	At admission At admission and discharge Within 1 wk from start of CR
5.	Kitamura M et al. (2019)^42^	Motor FIM, NYHA, RMI	At discharge
6.	Takada S et al. (2020)^29^	Length of time from admission to achievement of 40 m independent and unassisted walking, and length of time from admission to achievement of more than 50 m independent walking (equivalent of BI score of 15) NYHA class, BI	30 d At admission
7.	Yang J et al. (2021)^21^	6MWT	Before and after intervention
8.	Yamazaki Y et al. (2022)^25^	Exertional dyspnea after walking 10 m measured via VAS	Day 1 and day 3
9	Ahmad MA et al. (2023)^27^	BI, 6MWT	Baseline, and at discharge
10	Funato Y et al. (2022)^45^	NIL	NA
11.	Fraga IB et al. (2023)^36^	BI	At enrollment
12.	Ishibashi T et al. (2023)^37^	BI	Admission and discharge
Exercise training			
13.	Koifman E et al. (2014)^19^	6MWT	3 mo, 6 mo
14.	Mezzani A et al. (2014)^49^	6MWT	Day 1, 6, 12 and 30
15.	Delgado BM et al. (2020)^23^	LCADL, BI, and 6MWT	At discharge
16.	Shibata A et al. (2021)^38^	CPET	At admission and discharge
17.	Wang Y et al. (2022)^48^	NIL	NA
Early mobilization and exercise training			
18.	Kitamura M et al. (2017)^40^	Motor FIM, NYHA	At discharge (mean hospital stay of 17.3 d)
19.	Motoki H et al. (2019)^34^	BI	Pre and post CR
20.	Hamazaki N et al. (2020)^24^	6MWT NYHA	At baseline (5th day of training session) and discharge At admission and after 5 mo of CR
21.	Yokota J et al. (2021)^30^	BI SPPB, SAS, NYHA class	At admission and discharge (median of 24–44 d) At admission
22.	Kato M et al. (2021)^32^	Average daily rehabilitation time, and NCGG‐ADL BI, SPPB, usual gait speed, and leg strength	During acute phase rehabilitation At discharge
23.	Sakai T et al. (2021)^43^	CPET	2 mo and 5 mo
24.	Nemoto S et al. (2022)^39^	PMADL‐8	Stable phase
25.	Wu L et al. (2022)^28^	SPPB, ADL assessment, 6MWT	At baseline, before discharge, and 6 mo
26.	Kato M et al. (2022)^31^	BI, SPPB	At admission
27.	Yamazaki Y et al. (2022)^44^	Days to ambulate from admission BI, 6MWT	During hospital stay At discharge
28.	Nakaya Y et al. (2021)^26^	SPPB, QIS, usual gait speed Single leg‐stand time, TUG and 6MWT	At hospitalization and discharge or between hospital days 14 and 21 At discharge or between hospital days 14 and 21
29.	Enzan N et al. (2023)^35^	BI	At admission and discharge
30.	Kim J et al. (2023)^46^	NIL	NA

BI indicates Barthel index; CPET, cardiopulmonary exercise testing; DASI, Duke activity status index; FIM, functional independence measure; LCADL, London chest activity of daily living; NCGG‐ADL, National center for geriatrics and gerontology of activities of daily living; NYHA, New York Heart Association; PMADL‐8, performance measure for activities of daily living—8; QIS, Quadriceps isometric strength; RMI, Rivermead mobility index; SPPB, short physical performance battery; 6MWT, 6‐minute walk test; SAS, specific activity scale; TUG, timed up‐and‐go test; and VAS, visual analogue scale.

#### Early Mobilization

Four of the studies (4 of 12, 33.3%) followed the Japanese Circulation Society guidelines (JCS) for the intervention.^29^, ^41^, ^42^, ^45^, ^51^ Early mobilization was delivered at a dose of 7.25 sessions (3.2–25), 2 to 3 times/day, lasting for 2 to 48 days (Figure 2). Early mobilization was performed at low intensity, which was measured variably. Only 1 study estimated the patient’s response to the exercise loading test, to set the intensity and decide on progression.^45^ Along with gradual mobilization from supine to walking, additional exercises ranged from simple active range of motion exercises to resisted exercises. Endurance training was provided using a cycle ergometer in 50% (6 of 12) of the studies.^20^, ^29^, ^33^, ^36^, ^41^, ^42^ Progression was symptom‐limited; however, this was not elucidated explicitly (Table 2).

#### Early Exercise Training

Early exercise training was delivered at a median dose of 5.13 (4–60) sessions, 2 to 3 times/day for 5 to 180 days. Intensity was measured with the Borg’s rating of perceived exertion except for studies by Koifman et al. and Shibata et al., which measured exercise intensity using heart rate reserve and anaerobic threshold, respectively.^19^, ^38^ Early exercise training was mainly aerobic, and only 1 study provided aerobic and resistance exercises (Table 2).^19^

#### Early Mobilization with Exercise Training

More than half of the studies (8 of 13, 61.5%)^24^, ^26^, ^30^, ^31^, ^32^, ^35^, ^43^, ^46^ used the JCS guidelines to administer early mobilization with exercise training. It was delivered 2 to 5 times/week at a median dose of eight sessions (4.7–21), lasting between 5 days and 5 months. Interestingly, 10 of 13 (76.9%) studies^24^, ^26^, ^28^, ^31^, ^32^, ^34^, ^35^, ^40^, ^43^, ^46^ reported exercise training at moderate intensity. An exception was the study by Yokota et al., which offered a low‐intensity intervention throughout.^30^ The intensity of aerobic exercise was measured using Borg’s rating of perceived exertion, target heart rate, or heart rate reserve, and for resistance exercise, a percentage of one repetition maximum. Only 1 study provided a multi‐domain exercise training program which included balance training.^26^ The progression of exercises was mainly individualized except for 2 studies, in which 1 study progressed on the basis of stability of the clinical condition^39^ and the other progressed based on the mobility status of the patient.^28^

### Monitoring Criteria and Safety Checklist

Five studies^29^, ^33^, ^41^, ^42^, ^45^ on early mobilization, 2 studies^38^, ^48^ on exercise training, and 9^24^, ^28^, ^30^, ^31^, ^32^, ^35^, ^39^, ^40^, ^46^ studies on early mobilization with exercise training used a structured safety criterion while mobilizing patients with AHF. Among them, 68.7% (11 of 16)^24^, ^29^, ^30^, ^31^, ^32^, ^35^, ^41^, ^42^, ^43^, ^45^, ^46^ referred to the JCS guidelines^51^, ^52^ for safety criteria (Table 1). The existing safety criteria assess clinical stability, except for 1 study^23^ which determined safety by measuring the response of vital signs to exercise. Furthermore, these studies used the safety criteria to initiate an exercise session, except for 2 studies^27^, ^33^ which used a safety criterion to decide progression of an exercises. Only 3 studies used a termination criterion to stop the exercise training session, as outlined in the Table S3.^25^, ^27^, ^48^

## Discussion

Critical care cardiology has now evolved to propose early mobilization as one of the many requirements to ensure safety and quality of care.^53^ Hence, this review maps the evidence on early exercise‐based intervention during the recovery phase of AHF, from admission through the early post‐hospitalization period. This review identified 3 key findings: (i) most studies originated from Japan, (ii) the majority were observational in design, and (iii) early, low‐ to moderate‐intensity exercise‐based interventions were initiated as early as 4 days after admission, with a median of eight sessions per patient.

### Characteristics of Study Design

The predominance of observational studies parallels the research trajectory of early mobilization in the intensive care unit (ICU).^54^ The predominance of observational designs (Table 1) could be attributed to the complexity and critical clinical nature of AHF, which limits the feasibility of well‐designed RCTs in the cardiac ICU.^55^ Guidelines based on lower grades of evidence (Level C) can potentially reduce clinical adoption due to uncertainty among clinicians. Hence, novel study designs such as registry‐based RCTs, cluster trials, step wedge RCTs^7^ or interrupted time series,^7^ may offer solutions suitable for cardiac critical care settings.^55^

Skeletal muscle dysfunction plays a vital role in exercise intolerance in HF^56^ and similar to early mobilization studies in critical care,^54^, ^57^ included studies assessed both lower limb endurance (2‐minute step test and 30‐second chair stand)^47^ and strength (quadriceps isometric strength)^26^ in patients with AHF.

### Geographical Distribution

The concentration of studies in Japan may reflect both demographic trends—an aging heart failure population—and rehabilitation practices promoting early initiation of CR^52^ Japan supports early initiation of exercise‐based interventions, ie, in the recovery stage itself, to improve outcomes and uptake of CR services^51^, ^52^ Globally, CR faces inequality and is challenged by multi‐layered barriers, with socioeconomic factors being one of the significant barriers to CR adoption and adherence in LMICs.^58^, ^59^, ^60^ Furthermore, differences in research priorities between higher‐income countries and LMICs may also explain the uneven geographic distribution. Nevertheless, this review is limited by the exclusion of foreign language studies and likely to have contributed to the observed geographic skewness.

Substantial regional differences in the clinical and demographical characteristics of HF participants in LMIC.^61^, ^62^, ^63^, ^64^ Compared with higher‐income countries, patients in LMICs tend to be younger, with greater comorbidities, and variability in medication access and adherence—all of which may influence the efficacy of early rehabilitation.^62^, ^63^, ^65^ Evidence from trials such as EVEREST highlights that geographic factors modulate both morbidity and mortality outcomes.^66^ Regional disparities in heart failure profiles and the existing financial burden incurred by AHF further emphasize this gap.^2^, ^67^

### Timing and Initiation of Early Exercise‐Based Interventions

Early mobilization is known to improve functional capacity, walking distance within the hospital, muscle strength, and quality of life.^57^ In this review, initiation occurred within a median of 4 days after admission—later than typical ICU definitions of “early” mobilization—reflecting clinical caution regarding safety and hemodynamic stability.^68^ Yet, inconsistent initiation of early mobilization requires attention, as immobility during the first week of admission leads to muscle atrophy, especially among the critically ill.^69^ Moreover, a key limitation of this review is the exclusion of studies that initiated exercise‐based interventions at hospital discharge or within the early post‐discharge period—common time points for CR enrollment in AHF populations.^7^

Studies on early mobilization for critically ill patients have well‐defined indicators to initiate early mobilization.^70^, ^71^, ^72^ However, most studies in this review lacked a clear definition of the initiation criteria and initiation time. It was identified in a previous review on patients with AHF as well.^7^ In this review, it was also seen that most studies lacked standardized definitions of “hemodynamic stability” relying on health care provider’s discretion to determine readiness. The absence of uniform initiation criteria or structured referral protocols limits reproducibility and timely implementation. It reaffirms the need to establish initiation criteria or physician referral criteria to define the ideal time for early initiation of exercise‐based intervention during the hospitalization period. The development of standardized criteria could enable earlier initiation, faster progression to outpatient CR, and improved patient engagement following discharge.

### Exercise Prescription

The comprehensive nature of exercise training in our review aligned with JCS guidelines^51^ and REHAB‐HF.^9^ The subjectivity of Phase I CR makes it challenging to measure the optimal dose.^73^ This review identified a median of eight sessions provided to patients recovering from AHF, which is well above the sub‐optimal dose of 6 sessions (1 session per week×6 weeks).^74^ Training intensities were low to moderate and similar to the exercise prescription for chronic HF participants.^13^, ^56^ Interestingly, low‐intensity early mobilization facilitated transition to moderate intensity exercise training, resulting in an exercise dose equivalent to phase II CR.^75^

However, heterogeneity in exercise prescription—indicated by the wide confidence interval of exercise dose—and substantial variability in intensity assessment, was similar to studies on critically ill.^57^, ^76^ This can in turn lead to heterogeneity in clinical practice and/or overly cautious approaches that hinder the initiation of early exercise training.

### Monitoring Criteria and Safety Checklist

Safety remains a key determinant of the feasibility of early mobilization. An adverse event rate of 4% to 11% has been reported in studies on CR in patients with AHF, underscoring the need for structured safety and termination criteria.^7^, ^13^ Our review revealed a lack of structured safety and termination criteria, which could be a potential barrier to early mobilization.^68^ A similar finding was noted in a review of patients with AHF.^7^ It can delay the initiation of early mobilization, as supported by the wide CI in the initiation time (2.8–5.5 days) in our review.

The literature has shown that large consensus‐based studies are working towards ensuring the safety of ICU patients.^71^, ^72^ Furthermore, algorithms with specific criteria could facilitate the early initiation (28) and safe administration^68^ of exercise‐based interventions. It can guide various health care professionals in making evidence‐based decisions and ensure the uniform implementation of early exercise training, regardless of the setting.

### Research Gaps and Future Perspectives

The current evidence is limited by the scarcity of high‐quality RCTs and the lack of data from LMICs. Thus, higher‐income countries and upper middle income countries could consider collaborations with LMIC by undertaking large‐scale, multi‐centric trials. Furthermore, the uniformity of the initiation time, methods for measuring the intensity, monitoring criteria, and dose–response relationships are other areas of future research.

From a translational standpoint, adopting alternative CR delivery models^60^ to increase scalability and a roadmap with standardized checklists to facilitate early and safe mobilization during hospitalization is required. The use of multidisciplinary care teams to identify a suitable time to initiate early CR could also be incorporated. This could be achieved through expert consensus with multiple stakeholders, including patient and public involvement and engagement. Pragmatic trials using implementation frameworks^77^, ^78^, ^79^ can be used to address barriers to adoption, ensuring that early CR becomes an integral component of AHF recovery pathways.^5^, ^80^

In this effort, the authors developed a mobilization algorithm that incorporates multiple criteria and troubleshooting pathways, established through a Delphi consensus, and trained physiotherapists to facilitate the safe and standardized delivery of early mobilization and exercise training in patients recovering from acute heart failure.^81^ Discussion of this algorithm is beyond the scope of the present review.

## Conclusions

Early exercise‐based interventions were comprehensive, multimodal, of low to moderate intensity, and were initiated 4 days after admission. The literature, although rapidly growing, remains rudimentary in design and limited in LMIC settings. Standardized criteria for the initiation, referral, progression, and termination of early exercise interventions, along with the training of health care professionals, are needed to ensure safety and consistency in clinical practice. Collaborative, multidisciplinary approaches and pragmatic multicentric trials are essential to translate early CR into routine care across diverse health care settings.

## Sources of Funding

None.

## Disclosures

AS is a principal investigator on a grant funded by Heart failure Association of India (HFAI grant no. HFAI/RG/2022/1) and is currently funded by the Dr TMA Pai scholarship, MAHE, Manipal. HS—Received funding from the Indian Council of Medical Research, Governemnt of India. PJ—Supported by the Wellcome Trust through a Wellcome Trust/DBT India Alliance Clinical and Public Health Senior Fellowship (IA/CPHS/20/1/505229). Funding was received from the National Institute of Health, USA (5R01HL125442‐05), the National Health and Medical Research Council‐ Australia (1 160 283 and 1 169 766), the Medical Research Council—UK (MR/T037822/1), the Indian Council of Medical Research, Government of India, and the National Institute of Health Care Research, UK (NIHR204871 and NIHR205540). ASB—Received funding from the Indian Council of Medical Research, Govt of India and Science and Engineering Research Board (now known as the Anusandhan National Research Foundation), Govt. of India. RP resolved conflicts that arose during the screening of data and contributed to the interpretation of the data and revision of the manuscript. RP approves all the revisions of the manuscript and is accountable for the author’s own contribution. HS contributed to data interpretation and has substantially revised the manuscript. HS approves all the revision of the manuscript and is accountable for author’s own contribution. JP contributed to data interpretation and has substantially revised the manuscript. JP approves all the revisions of the manuscript and is accountable for author’s own contribution. MP contributed to data interpretation and has substantially revised the manuscript. MP approves all the revisions of the manuscript and is accountable for author’s own contribution. ASB contributed to the conceptualizing of the idea, designing of the work, screening of the data, and data interpretation. ASB has substantially revised the manuscript. ASB approves all the revisions of the manuscript and is accountable for author’s own contribution.

## Supporting information

Tables S1–S3
Figure S1
