# Supplementary material for: Mapping of Early Exercise‐Based Interventions for Patients Recovering From Acute Heart Failure: A Scoping Review
Source: J Am Heart Assoc. 2026 Feb 11;15(4):e045954. doi: 10.1161/JAHA.125.045954 (PMC13055831; doi:10.1161/JAHA.125.045954)

Supplemental Material

Table S1: Preferred Reporting Items for Systematic reviews and Meta-Analyses extension for Scoping Reviews (PRISMA-ScR) Checklist

SECTION	ITEM	PRISMA-ScR CHECKLIST ITEM	REPORTED ON PAGE #
**TITLE**			
Title	1	Identify the report as a scoping review.	1
**ABSTRACT**			
Structured summary	2	Provide a structured summary that includes (as applicable): background, objectives, eligibility criteria, sources of evidence, charting methods, results, and conclusions that relate to the review questions and objectives.	1-2
**INTRODUCTION**			
Rationale	3	Describe the rationale for the review in the context of what is already known. Explain why the review questions/objectives lend themselves to a scoping review approach.	3
Objectives	4	Provide an explicit statement of the questions and objectives being addressed with reference to their key elements (e.g., population or participants, concepts, and context) or other relevant key elements used to conceptualize the review questions and/or objectives.	3
**METHODS**			
Protocol and registration	5	Indicate whether a review protocol exists; state if and where it can be accessed (e.g., a Web address); and if available, provide registration information, including the registration number.	4
Eligibility criteria	6	Specify characteristics of the sources of evidence used as eligibility criteria (e.g., years considered, language, and publication status), and provide a rationale.	4
Information sources*	7	Describe all information sources in the search (e.g., databases with dates of coverage and contact with authors to identify additional sources), as well as the date the most recent search was executed.	4
Search	8	Present the full electronic search strategy for at least 1 database, including any limits used, such that it could be repeated.	Supplementary material, Table S2
Selection of sources of evidence†	9	State the process for selecting sources of evidence (i.e., screening and eligibility) included in the scoping review.	4-5
Data charting process‡	10	Describe the methods of charting data from the included sources of evidence (e.g., calibrated forms or forms that have been tested by the team before their use, and whether data charting was done independently or in duplicate) and any processes for obtaining and confirming data from investigators.	5
Data items	11	List and define all variables for which data were sought and any assumptions and simplifications made.	5
Critical appraisal of individual	12	If done, provide a rationale for conducting a critical appraisal of included sources of evidence; describe	NA

SECTION	ITEM	PRISMA-ScR CHECKLIST ITEM	REPORTED ON PAGE #
sources of evidence§		the methods used and how this information was used in any data synthesis (if appropriate).	
Synthesis of results	13	Describe the methods of handling and summarizing the data that were charted.	5
**RESULTS**			
Selection of sources of evidence	14	Give numbers of sources of evidence screened, assessed for eligibility, and included in the review, with reasons for exclusions at each stage, ideally using a flow diagram.	Supplementary material, Figure S1
Characteristics of sources of evidence	15	For each source of evidence, present characteristics for which data were charted and provide the citations.	5-9
Critical appraisal within sources of evidence	16	If done, present data on critical appraisal of included sources of evidence (see item 12).	NA
Results of individual sources of evidence	17	For each included source of evidence, present the relevant data that were charted that relate to the review questions and objectives.	5-9
Synthesis of results	18	Summarize and/or present the charting results as they relate to the review questions and objectives.	5-9
**DISCUSSION**			
Summary of evidence	19	Summarize the main results (including an overview of concepts, themes, and types of evidence available), link to the review questions and objectives, and consider the relevance to key groups.	9-12
Limitations	20	Discuss the limitations of the scoping review process.	11
Conclusions	21	Provide a general interpretation of the results with respect to the review questions and objectives, as well as potential implications and/or next steps.	12-13
**FUNDING**			
Funding	22	Describe sources of funding for the included sources of evidence, as well as sources of funding for the scoping review. Describe the role of the funders of the scoping review.	13

JBI = Joanna Briggs Institute; PRISMA-ScR = Preferred Reporting Items for Systematic reviews and Meta-Analyses extension for Scoping Reviews.
* Where *sources of evidence* (see second footnote) are compiled from, such as bibliographic databases, social media platforms, and Web sites.
† A more inclusive/heterogeneous term used to account for the different types of evidence or data sources (e.g., quantitative and/or qualitative research, expert opinion, and policy documents) that may be eligible in a scoping review as opposed to only studies. This is not to be confused with *information sources* (see first footnote).
‡ The frameworks by Arksey and O'Malley (6) and Levac and colleagues (7) and the JBI guidance (4, 5) refer to the process of data extraction in a scoping review as data charting.
§ The process of systematically examining research evidence to assess its validity, results, and relevance before using it to inform a decision. This term is used for items 12 and 19 instead of "risk of bias" (which is more applicable to systematic reviews of interventions) to include and acknowledge the various sources of evidence that may be used in a scoping review (e.g., quantitative and/or qualitative research, expert opinion, and policy document).
From: Tricco AC, Lillie E, Zarin W, O'Brien KK, Colquhoun H, Levac D, et al. PRISMA Extension for Scoping Reviews (PRISMAScR): Checklist and Explanation. Ann Intern Med. 2018;169:467–473. doi: 10.7326/M18-0850.

Table S2: Search strategy

PubMed:

**Search no.**	**Search strategy**
#1	"acute heart failure"[All Fields]
#2	"acute decompensated heart failure"[All Fields]
#3	"ADHF"[All Fields]
#4	"acute cardiac insufficiency"[All Fields]
#5	“heart failure"[All Fields]
#6	"heart failure, diastolic"[MeSH Terms]
#7	"heart failure, systolic"[MeSH Terms]
#8	#1 OR #2 OR #3 OR #4 OR #5 OR #6 OR #7
#9	"cardiac rehabilitation"[All Fields]
#10	“exercise training"[All Fields]
#11	“"early mobilization"[All Fields]
#12	"cardiac rehabilitation"[MeSH Terms]
#13	"exercise"[MeSH Terms]
#14	"early ambulation"[MeSH Terms]
#15	#9 OR #10 OR #11 OR #12 OR #13 OR #14
#16	#18 AND #15

Scopus

**Search no.**	**Search strategy**
#1	acute heart failure decompensation
#2	acute heart failure
#3	#1 OR #3
#4	early mobilization
#5	cardiac rehabilitation
#6	ambulation
#7	exercise training
#8	#4 OR #5 OR #6 OR #7
#9	#3 AND #8
#10	Title, abstract, keywords: acute heart failure
#11	#9 AND #10

Web of Science

Search no.	Search strategy
#1	ALL=("acute heart failure*")
#2	ALL=("acute decompensated heart failure")
#3	ALL=("decompensat* heart failure")
#4	ALL=("systolic heart failure")
#5	ALL=("diastolic heart failure")
#6	ALL=("heart failure")
#7	ALL=("acute cardiac insufficiency")
#8	#1 OR #2 OR #3 OR #4 OR #5 OR #5 OR #6 OR #7
#9	ALL=("early ambulation")
#10	ALL=("early mobilization")
#11	ALL=("exercise therapy")
#12	ALL=("exercise training")
#13	ALL=("cardiac rehabilitation")
#14	ALL=("phys* therap*")
#15	ALL=("physical rehabilitation")
#16	#9 OR #10 OR #11 OR #12 OR #13 OR #14 OR #15
#17	#8 AND #16
#18	Document type: Article
#19	#17 AND #18

EMBASE + MEDLINE

Search no.	Search strategy
#1	'acute heart failure'/exp/mj
#2	'acute heart failure syndrome'
#3	'acute heart failure'
#4	#1 OR #2 OR #3
#5	'exercise'/exp/mj
#6	exercise
#7	'heart rehabilitation'/exp/mj
#8	mobilization
#9	'mobilization'/exp/mj
#10	'physical therapy'
#11	#5 OR # 6 #7 OR #8 OR #9 OR #10
#12	Filters: ([article]/lim OR [article in press]/lim) AND [humans]/lim
#13	#4 AND #12
#14	#13 AND #14

Table S3: Termination criteria as reported by the included studies

Sl. No.	Author, year	Termination criteria
1.	Yamazaki Y et al., 2022	(1) New HF symptoms<br>(2) Increased arrhythmias, or rhythm changes to atrial fibrillation<br>(3) Increased heartbeats (>30 beats/min)<br>(4) A decrease in percutaneous oxygen saturation below 90%<br>(5) Excessive blood pressure changes<br>(6) Ischemic ST changes
2.	Wang Y et al., 2023	(1) Chest pain, palpitations, dyspnea, sweating, and other obvious discomfort symptoms<br>(2) ECG showed frequent ventricular tachycardia, atrial tachycardia, atrial fibrillation, and other malignant arrhythmias<br>(3) Systolic blood pressure did not increase but decreased by 10 mmHg or more, or systolic blood pressure elevated by 180 mmHg<br>(4) The patient requested to stop.
3.	Ahmad AM et al., 2023	(1) Symptoms or signs suggesting postural hypotension<br>(2) Intolerance to physical activity<br>(3) Poor central/peripheral perfusion such as dizziness, blurred vision, confusion, severe breathlessness or fatigue, chest pain, palpitations, leg cramp, cold sweating, pallor, >4% decrease in O2 saturation, cyanosis, or exaggerated heart rate response

Figure S1: PRISMA flow-diagram

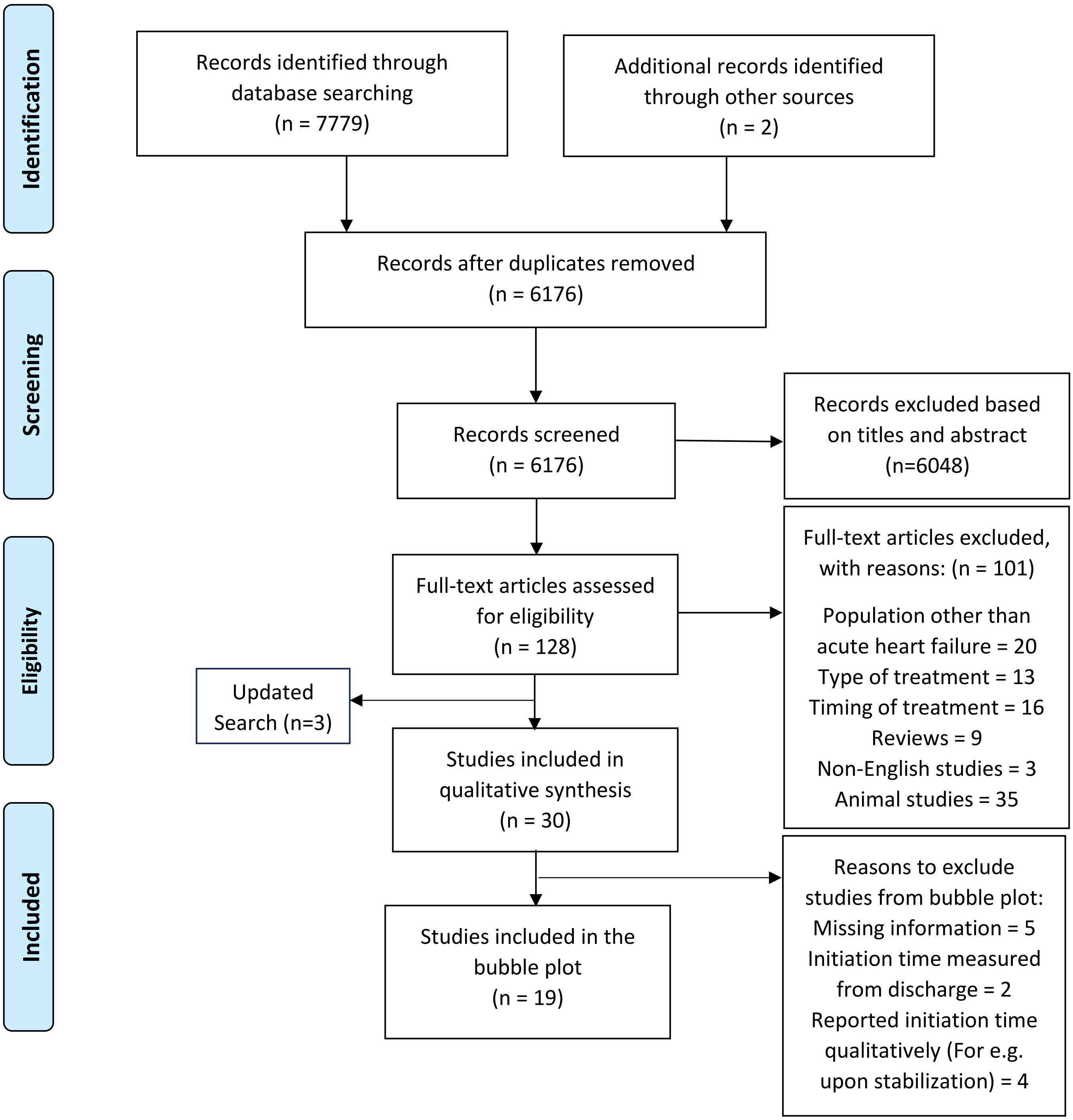

Supplement: Supplementary file 1 — Tables S1–S3 Figure S1 [file JAH3-15-e045954-s001.pdf]
